# Genus *Aconitum* (Ranunculaceae) in the Ukrainian Carpathians and adjacent territories

**DOI:** 10.3897/BDJ.11.e98828

**Published:** 2023-01-19

**Authors:** Andriy Novikov, Oleh Prylutskyi

**Affiliations:** 1 State Museum of Natural History of the NAS of Ukraine, Lviv, Ukraine State Museum of Natural History of the NAS of Ukraine Lviv Ukraine; 2 V. N. Karazin Kharkiv National University, Kharkiv, Ukraine V. N. Karazin Kharkiv National University Kharkiv Ukraine

**Keywords:** Occurrence, herbarium material, phytodiversity, mountain flora

## Abstract

**Background:**

The dataset represents a comprehensive collection of occurrence records concerning the genus *Aconitum* (Ranunculaceae) in the Ukrainian Carpathians and adjacent territories. It is based primarily on the results of critical revision of the main herbarium collections of the Carpathian region (i.e. LW, LWS, LWKS, KRA, KRAM, CHER, KW, UU and KWHU). Besides this, the dataset contains the data parsed (and taxonomically revised) from the published materials and other available sources (e.g. Karel Domin's Card Index).

**New information:**

In total, 2,280 occurrence records of the genus *Aconitum* representatives distributed in the Ukrainian Carpathians were published.

## Introduction

The Ukrainian Carpathians are one of the principal centres of floristic diversity and endemism in Ukraine, serving as a home for over 2500 plant species (Chopyk and Fedoronchuk 2015). Amongst them, many species of vascular plants are rare, endemic or have very limited distribution (Tasenkevich 2003, Tasenkevich 2014). In particular, the genus *Aconitum* L., one of the problematic taxonomical groups of the vascular plants, has a local centre of its distribution and diversity in the Carpathians (Mitka 2003, Mitka et al. 2007, Mitka 2014). Specifically, in the Ukrainian part of the Carpathian Mountains, 21 species of *Aconitum* and infraspecific taxa are distributed. From this number, ten *Aconitum* species and infraspecific taxa are threatened (Onyshchenko et al. 2022) and 11 are endemic or sub-endemic (Novikoff et al. 2016, Kliment et al. 2016). Taking into account such a high level of rarity and endemism, biogeography studies of the genus *Aconitum* can be useful to reveal the hotspots and stress the hypotheses of distribution patterns of other vascular plants in certain regions (Wang et al. 2009, Novikov and Mitka 2020, Wani et al. 2022). Data on distribution are also important for threat assessment of rare representatives of the genus *Aconitum* and other groups of vascular plants (Hamor et al. 2009,Turis et al. 2014, Agnihotri et al. 2015, Novikoff et al. 2016).

For all of these investigations, initial distribution information, based on herbarium material and field observations, is required. Access to Ukrainian herbarium collections has remained restricted for the last few years due to pandemic limitations (Baldini 2020, Cota-Sánchez 2020, Shiyan 2021). In the context of the current situation (i.e. the war and accompanying circumstances), Ukrainian collections will remain unavailable for an unexpectedly long period and can be damaged or even lost (Mosyakin and Shiyan 2022). At the same time, most of the available publications are Cyrillic and, hence, difficult to use by foreigners. Therefore, we believe that creating open-access datasets that follow recognised biodiversity information standards, as well as providing a translation of Cyrillic information and georeferencing of reported occurrences, is an important step to make biodiversity data from Ukraine accessible and usable for scientists worldwide.

## General description

### Purpose

The purpose of creating this dataset was to gather and georeference all available data on the distribution of representatives of the genus *Aconitum* in the Ukrainian Carpathians and adjacent territories and to make this dataset freely and readily available through the GBIF facilities. Online publication of such datasets ensures the broad application of biodiversity data from Ukraine even in the case of limited access to collections, their loss or damage.

## Sampling methods

### Study extent

The dataset contains information on 2,280 occurrences of *Aconitum* species and infraspecific taxa from the Ukrainian Carpathians and adjacent territories (Novikov 2022).

### Sampling description

Initially, the working list of 28 species and infraspecific taxa of the genus *Aconitum* that are potentially distributed in the Ukrainian Carpathians was created following the recent taxonomy (Mitka 2003, Novikoff and Mitka 2011). After that, the working identification key for these taxa has been created. All herbarium specimens of monkshoods from the Ukrainian Carpathians deposited in leading herbaria in Lviv (LW, LWS and LWKS), Uzhgorod (UU), Chernivtsi (CHER), Kyiv (KW) and Cracow (KRA and KRAM) were then critically revised using the created indentification key. As a result, the dataset containing the raw data extracted from the processed herbarium labels was created. Later this dataset has been completed with the data from available published sources and also supported by georeference information with an indication of the precision level of coordinates identification. The coordinates of the occurrences were extracted and verified manually, using the OpenStreetMap (OpenStreetMap contributors 2022) and QGIS (QGIS Development Team 2022) services. Finally, the dataset has been completed with the data parsed from the Karel Domin's Card Index, deposited at the Institute of Botany of SAS in Bratislava. Due to the high risk of misidentification of *Aconitum* taxa, doubtful or incomplete reports (e.g. without information allowing the clear identification of the reported specimen at least to the rank of the species) were avoided.

### Quality control

The data were cross-checked for correct identification and known distribution areas to delimit potential errors and outlets. In case of doubt or impossibility of a correct identification of species, the specimens were omitted from the analysis. In case of unusual reports from the new areas, such occurrences were critically revised or omitted due to a high risk of misidentification.

### Step description

The following steps were taken before working with the herbarium material:

1. Creation of a list of *Aconitum* species that are potentially distributed in the Ukrainian Carpathians and adjacent territories;
2. Clarification of the supraspecific and infraspecific taxonomy and morphology of the listed species to delimit similar and phylogenetically related taxa that potentially can be misidentified or confused;
3. Creation of the identification key with indication of similar taxa;
4. Creation of a working checklist with the nomenclatural synonyms of selected *Aconitum* species.

The following steps were taken during the work with herbarium materials:

1. Photo capture of herbarium vouchers of selected species;
2. Taxonomic revision of specimens following recent taxonomy;
3. Parsing and databasing the information (i.e. locality, collector, date and other relevant data) from the labels following the DarwinCore standard;
4. Translation of Cyrillic (i.e. Ukrainian and Russian) data from labels into English;
5. Georeferencing and verification of localities;
6. Quality check applying OpenRefine.

The following steps were taken during the work with literature sources:

1. Verification of the authors on their authority;
2. Extraction of reported data to the dataset following the DarwinCore standard;
3. Translation of Cyrillic (i.e. Ukrainian and Russian) data into English;
4. Georeferencing and verification of localities;
5. Quality check applying OpenRefine.

## Geographic coverage

### Description

**Figure 1.**
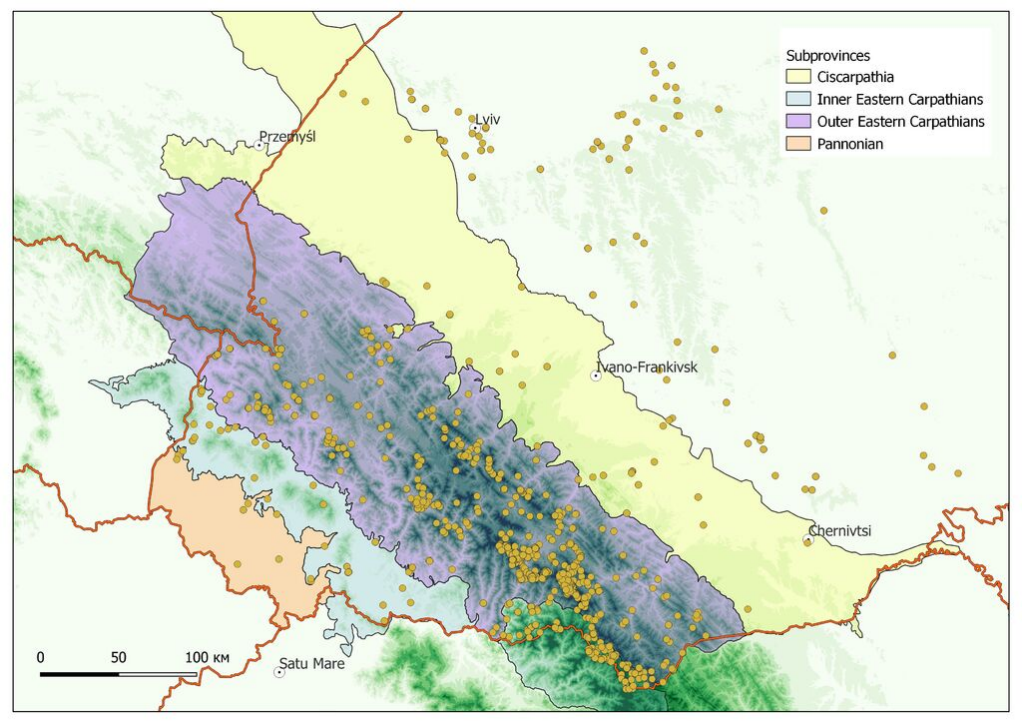
General distribution of georeferenced occurrences of the genus *Aconitum* in the Ukrainian Carpathians and adjacent territories.

The occurrences from the Ukrainian Carpathians and adjacent territories were considered (Fig. 1). Most of the databased occurrences are scattered in the region of the Ukrainian Carpathians. However, some analysed species (i.e. subendemic and non-endemic) have wider distribution and occur also in adjacent lowland territories. The occurrences of such species outside of the Ukrainian Carpathians were also taken into consideration to demonstrate their natural distribution patterns.

### Coordinates

47.8 and 51.5 Latitude; 22.7 and 25.9 Longitude.

## Taxonomic coverage

### Description

All analysed specimens and occurrence reports were identified to the lowest possible rank, usually to the level of species or subspecies. As a result, the dataset generally contains 40 *Aconitum* taxa, including 16 species and 24 infraspecific taxa. From this number, two species (i.e. *A.napellus* and *A.paniculatum*) do not correspond to the recent taxonomy of the genus (Mitka 2003) and cannot be unambiguously re-identified. The specimens identified as belonging to these two species (eight occurrences) were left in the dataset 'as is' with the hope of potential further clarifications. The dataset also contains 19 occasional occurrences of *A.besserianum*, *A.lycoctonum* and *A.pseudanthora* that are not represented in the flora of the Ukrainian Carpathians, but occur in adjacent territories.

### Taxa included

**Table:** 

Rank	Scientific Name	
species	*Aconitumanthora*	
subspecies	Aconitumanthorasubsp.anthora	
subspecies	Aconitumanthorasubsp.jacquinii	
species	*Aconitumbesserianum*	
species	*Aconitumbucovinense*	
species	*Aconitumcammarum*	
species	*Aconitumczarnohorense*	
species	*Aconitumdegenii*	
subspecies	Aconitumdegeniisubsp.degenii	
variety	Aconitumdegeniisubsp.degeniivar.intermedium	
species	*Aconitumfirmum*	
subspecies	Aconitumfirmumnothosubsp.fussianum	
subspecies	Aconitumfirmumsubsp.firmum	
subspecies	Aconitumfirmumsubsp.fissurae	
species	*Aconitumgayeri*	
species	*Aconitumlasiocarpum*	
subspecies	Aconitumlasiocarpumsubsp.kotulae	
subspecies	Aconitumlasiocarpumsubsp.lasiocarpum	
species	*Aconitumlycoctonum*	
subspecies	Aconitumlycoctonumsubsp.lycoctonum	
species	*Aconitummoldavicum*	
subspecies	Aconitummoldavicumnothosubsp.confusum	
subspecies	Aconitummoldavicumnothosubsp.porcii	
subspecies	Aconitummoldavicumnothosubsp.simonkaianum	
subspecies	Aconitummoldavicumsubsp.confusum	
subspecies	Aconitummoldavicumsubsp.hosteanum	
subspecies	Aconitummoldavicumsubsp.moldavicum	
species	*Aconitumnanum*	
species	*Aconitumnapellus*	
subspecies	Aconitumnapellussubsp.tauricum	
variety	Aconitumnapellussubsp.tauricumvar.nanum	
species	*Aconitumpaniculatum*	
species	*Aconitumpseudanthora*	
species	*Aconitumvariegatum*	
subspecies	Aconitumvariegatumsubsp.nasutum	
subspecies	Aconitumvariegatumsubsp.variegatum	

## Temporal coverage

**Living time period:** 1803-2019.

## Usage licence

### Usage licence

Creative Commons Public Domain Waiver (CC-Zero)

## Data resources

### Data package title

Genus Aconitum of the Ukrainian Carpathians and adjacent territories

### Resource link

https://doi.org/10.15468/n37j8x

### Alternative identifiers

https://www.gbif.org/dataset/91f04313-0699-41a7-87ef-2b0c13180778, https://ukraine.ipt.gbif.no/resource?r=aconitum_carpathians

### Number of data sets

1

### Data set 1.

#### Data set name

Genus Aconitum of the Ukrainian Carpathians and adjacent territories

#### Data format

Darwin Core

#### Character set

utf8

#### Download URL

https://www.gbif.org/dataset/91f04313-0699-41a7-87ef-2b0c13180778

#### Description

The tab-delimited CSV formatted dataset (Novikov 2022) has been created following Darwin Core standards and contains all available data on the distribution of the genus *Aconitum* representatives in the Ukrainian Carpathians and adjacent territories.

**Data set 1.** 

Column label	Column description
occurrenceID	An unique identifier for the Occurrence (as opposed to a particular digital record of the occurrence).
basisOfRecord	The specific nature of the data record, for example, preserved specimen or field observation.
collectionCode	Unique code of collection (e.g. herbarium) for the specimen deposited.
institutionCode	Unique code of institution (e.g. museum or herbarium) for the specimen deposited.
catalogNumber	An identifier for the record within collection.
scientificName	The full scientific name of taxon including at least the genus and species epithets and, in some cases, including the subspecies epithet.
taxonRank	The taxonomic rank of the most specific name in the scientificName.
recordedBy	A person, group or organisation responsible for recording the original Occurrence.
verbatimEventDate	The date of record as it appears in the original publication or specimen's label.
EventDate	The date during which an event (e.g. collection of the specimen, photographing of the plant or its registering in the field in any other way), occurred.
day	The day when occurrence was recorded.
month	The month when occurrence was recorded.
year	The year when occurrence was recorded.
fieldNumber	An identifier given to the specimen in the field by the collector.
identifiedBy	A list of names of people who assigned the Taxon to the subject,
dateIdentified	The date on which the subject was determined as representing a certain Taxon.
identificationRemarks	Comments or notes about the Identification.
decimalLatitude	The geographic latitude (in decimal degrees, using the spatial reference system given in geodeticDatum) of the geographic centre of a Location.
decimalLongitude	The geographic longitude (in decimal degrees, using the spatial reference system given in geodeticDatum) of the geographic centre of a Location.
coordinateUncertaintyInMetres	The horizontal distance (in metres) from the given decimalLatitude and decimalLongitude describing the smallest circle containing the whole of the Location.
geodeticDatum	The ellipsoid, geodetic datum or spatial reference system (SRS) upon which the geographic coordinates given in decimalLatitude and decimalLongitude are based. In our case, it is always WGS84.
verbatimElevation	The original description of the elevation (altitude, usually above sea level) of the Location.
countryCode	The standard code (ISO 3166-1-alpha-2) for the country in which the Location occurs.
country	The name of the country in which the Location occurs.
locality	The specific description of the place where the specimen was registered or collected.
verbatimLocality	The original textual description of the place where the specimen was registered or collected.
fieldNotes	The original text of notes taken in the field about the specimen by the collector.
associatedReferences	A list (concatenated and separated) of identifiers (publication, bibliographic reference, global unique identifier, URI) of literature associated with the Occurrence.
kingdom	The full scientific name of the kingdom in which the taxon is classified. In our case, it is always Plantae.
language	The language of the resource. In our case, herbarium labels contained information in different languages and sometimes different languages were even combined on a single label. To simplify the work with data, we indicated the languages applied for the data.
minimumElevationInMetres	The lower limit of the range of elevation (altitude, usually above sea level) in metres.
maximumElevationInMetres	The upper limit of the range of elevation (altitude, usually above sea level) in metres.
higherGeography	A list of generalised toponyms less specific than the information captured in the locality term (e.g. names of the mountain ridges, biogeographical zones, other natural areas etc.)
